# Epigenetic Regulation of Multiple Tumor-Related Genes Leads to Suppression of Breast Tumorigenesis by Dietary Genistein

**DOI:** 10.1371/journal.pone.0054369

**Published:** 2013-01-14

**Authors:** Yuanyuan Li, Huaping Chen, Tabitha M. Hardy, Trygve O. Tollefsbol

**Affiliations:** 1 Department of Biology, University of Alabama at Birmingham, Birmingham, Alabama, United States of America; 2 Center for Aging, University of Alabama at Birmingham, Birmingham, Alabama, United States of America; 3 Comprehensive Cancer Center, University of Alabama at Birmingham, Birmingham, Alabama, United States of America; 4 Nutrition Obesity Research Center, University of Alabama at Birmingham, Birmingham, Alabama, United States of America; 5 Comprehensive Diabetes Center, University of Alabama at Birmingham, Birmingham, Alabama, United States of America; King Faisal Specialist Hospital & Research center, Saudi Arabia

## Abstract

Breast cancer is one of the most lethal diseases in women; however, the precise etiological factors are still not clear. Genistein (GE), a natural isoflavone found in soybean products, is believed to be a potent chemopreventive agent for breast cancer. One of the most important mechanisms for GE inhibition of breast cancer may involve its potential in impacting epigenetic processes allowing reversal of aberrant epigenetic events during breast tumorigenesis. To investigate epigenetic regulation for GE impedance of breast tumorigenesis, we monitored epigenetic alterations of several key tumor-related genes in an established breast cancer transformation system. Our results show that GE significantly inhibited cell growth in a dose-dependent manner in precancerous breast cells and breast cancer cells, whereas it exhibited little effect on normal human mammary epithelial cells. Furthermore, GE treatment increased expression of two crucial tumor suppressor genes, *p21^WAF1^* (*p21*) and *p16^INK4a^* (*p16*), although it decreased expression of two tumor promoting genes, *BMI1* and *c-MYC*. GE treatment led to alterations of histone modifications in the promoters of *p21* and *p16* as well as the binding ability of the c-MYC–BMI1 complex to the *p16* promoter contributing to GE-induced epigenetic activation of these tumor suppressor genes. In addition, an orally-fed GE diet prevented breast tumorigenesis and inhibited breast cancer development in breast cancer mice xenografts. Our results suggest that genistein may repress early breast tumorigenesis by epigenetic regulation of *p21* and *p16* by impacting histone modifications as well as the BMI1-c-MYC complex recruitment to the regulatory region in the promoters of these genes. These studies will facilitate more effective use of soybean product in breast cancer prevention and also help elucidate the mechanisms during the process of early breast tumorigenesis.

## Introduction

Breast cancer is the most frequently diagnosed malignant neoplasm in women. Although the causes for initiation of breast carcinogenic procedures are not fully understood, considerable evidence has been put forward indicating that breast tumorigenesis involves complicated genetic and epigenetic abnormalities that include a large set of aberrant expression in tumor suppressor genes and oncogenes [1], [2]. While multiple gene expression abnormalities could attribute to germline mutations in high-penetrance cancer susceptibility genes including *BRCA1* and *TP53*, epigenetic aberration-induced gene expression changes also play a major role in initiating breast carcinogenic processes [3]–[6].

Epigenetic processes control gene expression through mechanisms that do not affect the primary DNA sequence such as mutation and deletion [7]–[9]. Epigenetic mechanisms mediate chromatin structure through regulation of DNA methylation, histone variants, RNA interference and posttranslational modifications, in which DNA methylation and histone modifications are recognized as the most important pathways for epigenetic control [8]–[10]. Besides their critical roles in promoting embryogenesis and early development, epigenetic events are critical in regulating the processes of carcinogenesis. Compared to the normal cells, aberrant epigenetic alterations occur within a larger context of chromatin in neoplastic cells, involving both losses and gains of DNA methylation as well as altered patterns of histone modifications [2], [7]. The best approach to better elucidate epigenetic impact on breast cancer initiation and progression is to understand epigenetic transcriptional repression of a growing list of candidate tumor suppressor genes. The silencing of gene expression is associated with abnormally dense accumulation of DNA methylation at the gene regulatory regions [7], [9]. For example, hypermethylation of the promoter of the tumor suppressor gene, *p16^INK4a^*, is frequently seen during early metaplastic progression in many human malignancies [11], [12]. In addition, altered histone modification profiles also contribute to transcriptional silencing of multiple tumor suppressor genes due to direct impact such as compacted chromatin structure and DNA replication failure as well as indirect effects involving recruitment of transcriptional suppressor complexes induced by chromatin changes [7], [8].

Although the precise molecular impact of epigenetic control on breast tumor initiation is only beginning to be elucidated, an abundance of clinical trials and experimental research have found that many pharmaceutical- or phytochemicals-derived compounds with epigenetic properties show promising preventive and therapeutic effects on breast cancer. Recently, the “epigenetic diet” has received extensive attention due to the abilities of these bioactive dietary compounds in prevention of various human cancers mediated by epigenetic events and dietary soybean genistein is one of the components of the epigenetic diet [13]–[15]. Genistein (GE) is a botanical isoflavone enriched in soybean products, such as soymilk and toufu [16]. Epidemiological studies have found that the incidence of breast cancer in Asian women who consumed GE-rich soy products as their traditional daily diet is much lower than American women suggesting that GE is a potent dietary chemopreventive compound against human breast cancers [17], [18]. Although GE exerts its anti-cancer properties through various mechanisms such as anti-oxidation and induction of apoptosis and differentiation, one potential mechanism that has recently received considerable attention is that GE may regulate gene transcription by modulating epigenetic events [19]–[21]. Supportive studies from our and other laboratories have shown that GE influences multiple aspects of epigenetic pathways including DNA methylation and histone modifications that facilitate reversal of aberrant epigenetic events leading to breast cancer prevention and therapy [22]–[24].

Current *in vitro* studies primarily focus on established cancer cell lines which have already undergone the processes of tumor development and progression. To observe the impact of GE on early events of breast cancer initiation, we applied an established cellular system in this study which mimics the process of early human breast tumorigenesis including different stages of transformed breast cells during breast cancer initiation [25], [26]. Therefore, by monitoring the epigenetic alteration of tumor-related genes in different stages of transformed breast cells, we could easily approach the detailed mechanisms for GE-induced chemoprevention in the early processes of breast cancer development.

In the present study, we observed expression changes and epigenetic modulations of several key tumor-related genes, including tumor suppressor genes, *p21^WAF1^* (*p21*) and *p16^INK4a^* (*p16*), and tumor promoting genes, *BMI1* and *c-MYC*, during early breast tumorigenesis *in vitro* and *in vivo*. Our studies reveal an important role of GE on epigenetic regulation of key tumor-associated genes that effectively reduces early tumor progression. Our findings indicate *p21*/*p16* and *BMI1*/*c-MYC* could serve as functional targets of dietary factor, GE, to inhibit early breast carcinogenesis and prevent breast cancer. This study helps to discover the potential mechanisms by which GE prevents breast cancer, and it may facilitate development of target gene therapy by using soybean product and other epigenetic modulators in future clinical practice.

## Materials and Methods

### Cell culture and cell treatment

**Figure 1:**
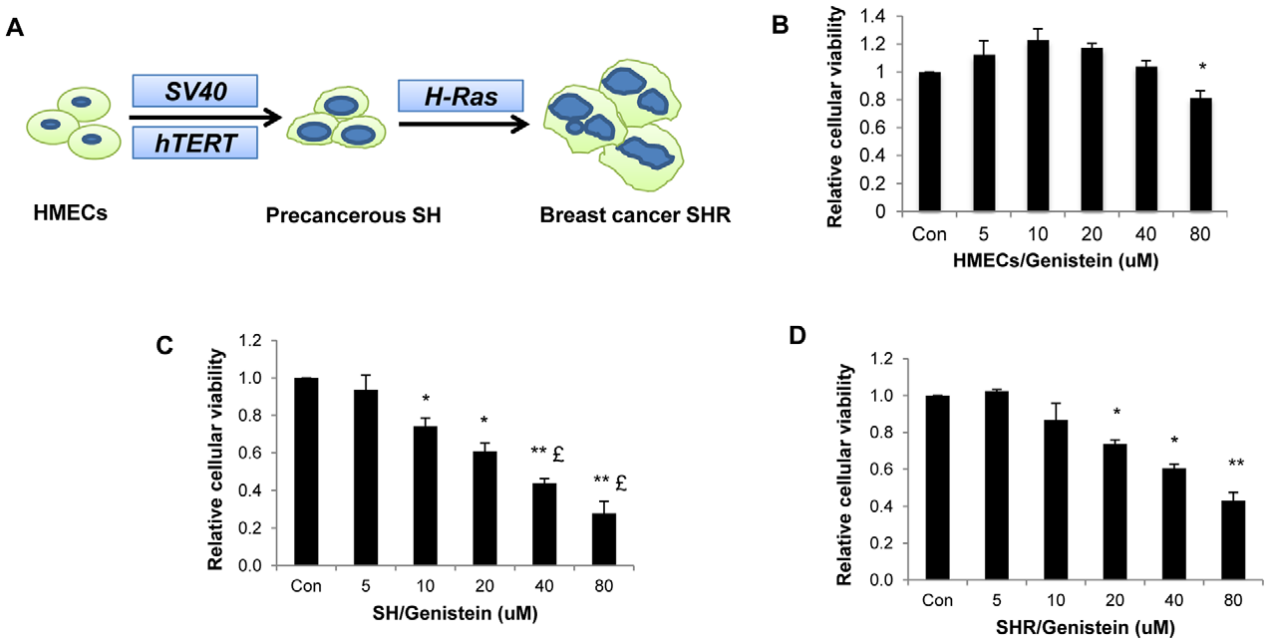
GE suppressed cellular growth in transformed breast cells. A, Schematic presentation of malignant transformation of the breast tumorigenesis cellular model. Normal HMECs stably transfected with *SV40, hTERT* and *h-RAS* to generate early transformed SH cells (precancerous cells) and completely transformed SHR cells (breast cancer cells). B, C and D, Cellular viability in response to various concentrations of GE treatment in HMECs (B), precancerous SH cells (C) and breast cancer SHR cells (D). Cells were plated in 96-well plates in triplicate and exposed to various concentrations of GE for up to 3 days. Cellular viability was measured by MTT assay. Data are in triplicate from three independent experiments and calibrated to levels in untreated samples. Columns, mean; Bars, SD; *, *P*<0.05, * * *P*<0.01, significantly different from control; £, *P*<0.05, significantly different from SHR cells with the corresponding treatment.

Normal human mammary epithelial cells (HMECs) were obtained from Lonza (Basel, Switzerland). Early transformed SH cells (precancerous cells; normal HMECs stably transfected with *SV40* and human telomerase reverse transcriptase, *hTERT*) and completely transformed SHR cells (breast cancer cells; SH cells except with added *H-Ras*) were established in our laboratory (Fig. 1A) [27]. An established breast cancer cell line, MDA-MB-231 cells, obtained from the American Type Culture Collection (ATCC) was used as a reference. HMECs were grown in serum-free Mammary Epithelial Growth Medium (MEGM) without sodium bicarbonate accompanied with MEGM SingleQuots (Lonza) at 37°C and 0.1% CO_2_. Breast cancer cells were grown in DMEM (Invitrogen, Carlsbad, CA) supplemented with 10% fetal bovine serum (Atlanta Biologicals, Lawrenceville, GA) and 1% penicillin/streptomycin (Mediatech, Herndon, VA) in a humidified environment of 5% CO_2_ and 95% air at 37°C. To evaluate the effect of genistein (GE) treatment, attached HMECs, SH and SHR cells were treated with various concentrations of GE (Sigma, St. Louis, MO) for 3 days. The medium with GE was replaced every 24 h for the duration of the experiment.

### MTT assay for cell viability and IC50

To determine the effects of GE on cell viability, aliquots of 2×10^3^ HMECs, SH and SHR cells were seeded in triplicate in 96-well plates and treated with the indicated compounds as described above. To determine dose-dependent inhibition rates and IC50 for GE, aliquots of SH, SHR and MDA-MB-231 cells were treated with titration of GE at concentrations of 0, 2.5, 5, 10, 20, 40, 80, 160, 200, 300 and 400 µM for 3 days. MTT solution was added to the medium to achieve a final concentration of 1 mg/ml. The cells were incubated at 37°C and dissolved in 100 µl DMSO after 4 h incubation. The absorbance of the cell lysates in DMSO solution was read at 570 nm by a microplate reader (Bio-Rad, Hercules, CA).

### Cell apoptosis analysis

Precancerous SH cells and transformed breast cancer SHR cells with 40 µM of GE treatment were collected and washed with cold p*hosphate* buffered saline (PBS). Cells were then used for apoptosis analysis with the Vybrant Apoptosis Assay kit #2 (Invitrogen). After fixation with the annexin-binding buffer, cells were stained both with Alexa Fluor Annexin V and propidium iodide (PI) according to the manufacturer's instructions. Flow cytometry analyses were performed on a Becton Dickinson FACSCalibur flow cytometer (Becton Dickinson, Franklin Lakes, NJ, USA). The fluorescence intensity of the viable cells was analyzed using CellQuest software.

### Animal models and dietary preparation

We used an orthotopic breast cancer mouse model in this study. Virgin female immunodeficiency Nu/Nu Nude mice (Crl:NU-*Foxn1nu*) were used for the xenograft breast cancer study. Nude mice at 4–6 weeks of age were obtained from Charles River Laboratories (Wilmington, MA). All the mice were housed in the Animal Resource Facility of the University of Alabama at Birmingham and were maintained under the following conditions: 12-h dark/12-h light cycle, 24±2°C temperatures, and 50±10% humidity.

Two designed diets were used in this study: control diet (phytoestrogen-free modified AIN-93G diet with 7% corn oil substituted for 7% soybean oil; TD. 95092; Harlan Teklad, Madison, WI) and GE diet (modified AIN-93G diet supplemented with 250 mg/kg genistein; TD. 00417; Harlan Teklad) [28]. The level of GE in this diet results in the animals being exposed to concentrations comparable with those received by humans consuming high-soy diets [29]. Harland Teklad supplied all diet ingredients except GE powder obtained from LKT Laboratories, St. Paul, MN.

### Animal experimental designs

After one week of acclimatization, Nu/Nu Nude mice were randomly divided into two groups (5 mice each) and administered either a control or GE diet as described above. Diets were provided from two weeks prior to injection and the mice continued to receive the corresponding experimental diets throughout the study. To determine the *in vivo* efficacy of GE in human transformed breast tumor xenografts, exponentially growing SHR cells at around 20 population doublings (PDs) were mixed at a 1∶1 ratio with Matrigel (Becton Dickinson). A 100 µl suspension containing 2×10^6^ cells was injected orthotopically into the mammary fat pad of each mouse.

Tumor diameters and body weight were measured weekly. Tumor volumes were measured by a caliper and estimated using the following formula: tumor volume (cm^3^)  =  (length × width^2^) ×0.523 [30]. The experiment was completed when the mean of tumor diameter in the control mice exceeded 1.0 cm following the guidelines of Institutional Animal Care and Use Committee at the University of Alabama at Birmingham. At the end of the experiment, the mice were sacrificed, primary tumors were excised and weighed. A tumor slice from each primary tumor tissue was carefully dissected and fixed in 10% buffer-neutralized formalin for histology and immunohistochemistry. Tumor specimens were snap frozen in liquid nitrogen for further studies such as RNA and protein extraction. All procedures with animals were reviewed and approved by the Institutional Animal Care and Use Committee at the University of Alabama at Birmingham (Animal Project Number: 110109327).

### Quantitative real-time PCR

Both precancerous SH and breast cancer SHR cells were cultured and treated as described above. Total RNA from cells or mice tumor tissues was extracted using the RNeasy kit (Qiagen, Valencia, CA) according to the manufacturer's instructions. Genes of interest were amplified using 1 µg of total RNA reverse transcribed to cDNA using the Superscript II kit (Invitrogen) with oligo-dT primer. In the real-time PCR step, PCR reactions were performed in triplicate and primers specific for *p16^INK4a^, p21^WAF1^, BMI1, c-MYC* and *glyceraldehyde-3-phosphate dehydrogenase* (*GAPDH*) provided by Integrated DNA Technologies were used for SYBR GreenER qPCR Supermix (Invitrogen) in a Roche LC480 thermocycler. Thermal cycling was initiated at 94°C for 4 min followed by 40 cycles of PCR (94°C, 15 s; 60°C, 30 s) and melting curve analysis. GAPDH was used as an endogenous control, and vehicle control was used as a calibrator. The relative changes of gene expression were calculated using the following formula: fold change in gene expression, 2^−ΔΔCt^  = 2^−{ΔCt (treated samples) – ΔCt (untreated control samples)}^, where ΔCt  =  Ct (test gene) – Ct (GAPDH) and Ct represents threshold cycle number.

### Western blot analysis

For western blot analysis, protein extracts were prepared by RIPA Lysis Buffer (Upstate Biotechnology, Charlottesville, VA) according to the manufacturer's protocol. Proteins (50 µg) were electrophoresed on a 10% SDS-polyacrylamide gel and transferred onto nitrocellulose membranes. Membranes were probed with antibodies to p16 (F-12; Santa Cruz Biotechnology, Santa Cruz, CA), p21 (C-19; Santa Cruz Biotechnology), BMI1(C-20, Santa Cruz Biotechnology) and c-MYC (A-14; Santa Cruz Biotechnology), respectively, then each membrane was stripped and reprobed with beta-actin antibody (13E5, Cell Signaling Technology, Boston, MA) as loading control. Molecular weight markers were run on each gel to confirm the molecular size of the immunoreactive proteins. Immunoreactive bands were visualized using the enhanced chemiluminescence detection system (Santa Cruz Biotechnology) following the protocol of the manufacturer.

### Immunohistochemical determination of tumor cell proliferation

Tumor sections (5 µm thick) were deparaffinized and rehydrated in a series of graded alcohols. Following rehydration, an antigen retrieval process was performed by placing the slides in 10 mmol/L sodium citrate buffer (pH 6.0) at 95°C for 20 min followed by 20-min cooling at room temperature. The sections were washed in PBS and nonspecific binding sites were blocked with 1% bovine serum albumin with 2% goat serum in PBS before incubating with anti-proliferating cell nuclear antigen (PCNA) (Cell Signaling Technology) for 2 h at room temperature. After washing with PBS, the sections were incubated with biotinylated secondary antibody for 45 min followed by horseradish peroxidase-conjugated streptavidin, washed in PBS, incubated with diaminobenzidine substrate, and counterstained with hematoxylin. Photographs of representative pictures were taken and the numbers of PCNA-positive cells were detected and counted using a light microscope. The results are presented as the number of positive cells ×100 divided by the total number of cells.

### Chromatin Immunoprecipitation (ChIP) Assay

Both precancerous SH and breast cancer SHR cells were treated with 40 µM GE for the indicated times. Approximately 2×10^6^ cells were cross-linked with a 1% final concentration of formaldehyde (37%, Fisher Chemicals, Fairlawn, NJ) for 10 min at 37°C. ChIP assays were performed with the EZ Chromatin Immunoprecipitation (EZ ChIP^TM^) assay kit according to the manufacturer's protocol (Upstate Biotechnology, Billerica, MA) as described previously [24], [31]. The epigenetic antibodies used in the ChIP assays were ChIP-validated acetyl-histone H3, acetyl-histone H4, trimethyl-histone H3-Lys4 (Me3H3K4), trimethyl-histone H3-Lys9 (Me3H3K9) and trimethyl-histone H3-Lys27 (Me3H3K27) from Upstate Biotechnology. ChIP-purified DNA was amplified by standard PCR using primers specific for the *p16* promoter yielding a 130 bp fragment: sense, 5′-TAGGAAGGTTGTATCGCGGAGG-3′ and anti-sense, 5′-CAAGGAAGGAGGACTGGGCTC-3′ as well as the *p21* promoter yielding a 249 bp fragment: sense, 5′-GGGGCGGTTGTATATCAGG and anti-sense, 5′-GTGAACGCAGCACACACC-3′. PCR amplification was performed using the 2×PCR Master Mix (Promega, Madison, WI) and the reaction was initiated at 94°C for 4 min followed by 30 cycles of PCR (94°C, 30 s; 56°C, 30 s; 72°C, 1 min), and extended at 72°C for 5 min. After amplification, PCR products were separated on 1.5% agarose gels and visualized by ethidium bromide fluorescence using Kodak 1D 3.6.1 image software (Eastman Kodak Company, Rochester, NY). Quantitative data were analyzed using the Sequence Detection System software version 2.1 (PE Applied Biosystems, Foster City, CA).

### Histone deacetylases (HDACs) and histone methyltransferases (HMTs) activity assay

Nuclear protein from cultured SH and SHR cells was extracted by using the nuclear extraction reagent (Pierce, Rockford, IL). The activities of HDACs (Active Motif, Carlsbad, CA) and HMT (Me3H3K4), HMT (Me3H3K9) and HMT (Me3H3K27) (Epigentek, Brooklyn, NY, USA) were performed according to the manufacturer's protocols as reported previously [31]. The enzymatic activities of HDACs and HMTs (Me3H3K4, Me3H3K9 and Me3H3K27) were detected by using a microplate reader at 450 nm.

### Statistical analyses

Microscopic immunohistochemical analysis of tissue sections was performed using an Olympus BX41 microscope fitted with a Q-color 5 Olympus camera. Results from Real-time PCR and ChIP assays were derived from at least three independent experiments. For quantification of ChIP products, Kodak 1D 3.6.1 image software was used. The protein levels were quantified by optical densitometry using ImageJ Software version 1.36b (http://rsb.info.nih.gov/ij/). Statistical significance between treatment and control groups was evaluated by one-way ANOVA followed by Tukey's test for multiple comparisons by using GraphPad Prism version 5.00 for Windows, GraphPad Software (www.graphpad.com). Dose-dependent inhibition rates and IC50 were analyzed by non-linear regression analysis (GraphPad Prism). Values were presented as mean ± SD and *P*<0.05 was considered significant.

## Results

### GE suppressed cellular growth in precancerous SH cells and breast cancer SHR cells without affecting growth of normal HEMCs

In current study, we introduced a well studied cellular cancer transformation system that closely mimics the process of early human breast tumorigenesis since we have extensive experience using this model for cancer research [27], [32], [33]. In this model, three defined genetic elements including *SV40, hTERT* and *H-RAS* were introduced into normal HMECs (Fig. 1A). This resulted in a neoplastic transformation and generation of different stages of human mammary cancer cells, including precancerous SH cells (transfected with *SV40* and *hTERT*) and transformed breast cancer SHR cells (transfected with all three genetic elements). This system has been verified for its successive genetic transfection and carcinogenic transformation by various experiments including protein expression, anchorage-independent colony formation and tumorigenesis in immunedeficient nude mice [26], [27]. Cell clones for precancerous SH cells and transformed breast cancer SHR cells used in this study were established in our laboratory at around 20 population doublings when experiments were initiated.

We initiated our study to determine the optimal concentration of GE that can inhibit cellular growth in breast cancer cells without affecting normal cell growth. We treated precancerous SH cells, breast cancer SHR cells and normal HEMCs with various concentrations of GE and measured cell viability under these treatments. As shown in Fig. 1, GE significantly inhibited cellular growth in precancerous SH cells and breast cancer SHR cells in a dose-dependent manner, especially in precancerous SH cells, suggesting that GE treatment may be more effective in prevention rather than treatment of breast cancer. IC50 values for precancerous SH cells and breast cancer SHR cells were 50.36 µM and 113.8 µM (Information S1), respectively, further indicating that GE treatment is much more effective in breast precancerous SH cells and GE may primarily exert its anti-cancer effect during the early stages of breast cancer development.

We also evaluated the potential toxicity of GE in normal breast HMECs and found that the tested concentrations of GE under 80 µM did not cause inhibitory effects on cell viability in HMECs indicating the treatment of GE under 80 µM is potentially safe. We noticed that 40 µM of GE treatment caused a more promising suppression of cell viability in breast cancer cells without affecting normal HEMCs compared to the effects under the other treatments of GE. This GE concentration is physiologically accessible by daily consumption of soybean product or a pharmaceutically available GE supplementary tablet [34]. We therefore considered a 40 µM of GE treatment as an optimal concentration in our study.

### GE-induced cellular apoptosis in precancerous SH cells and breast cancer SHR cells

**Figure 2:**
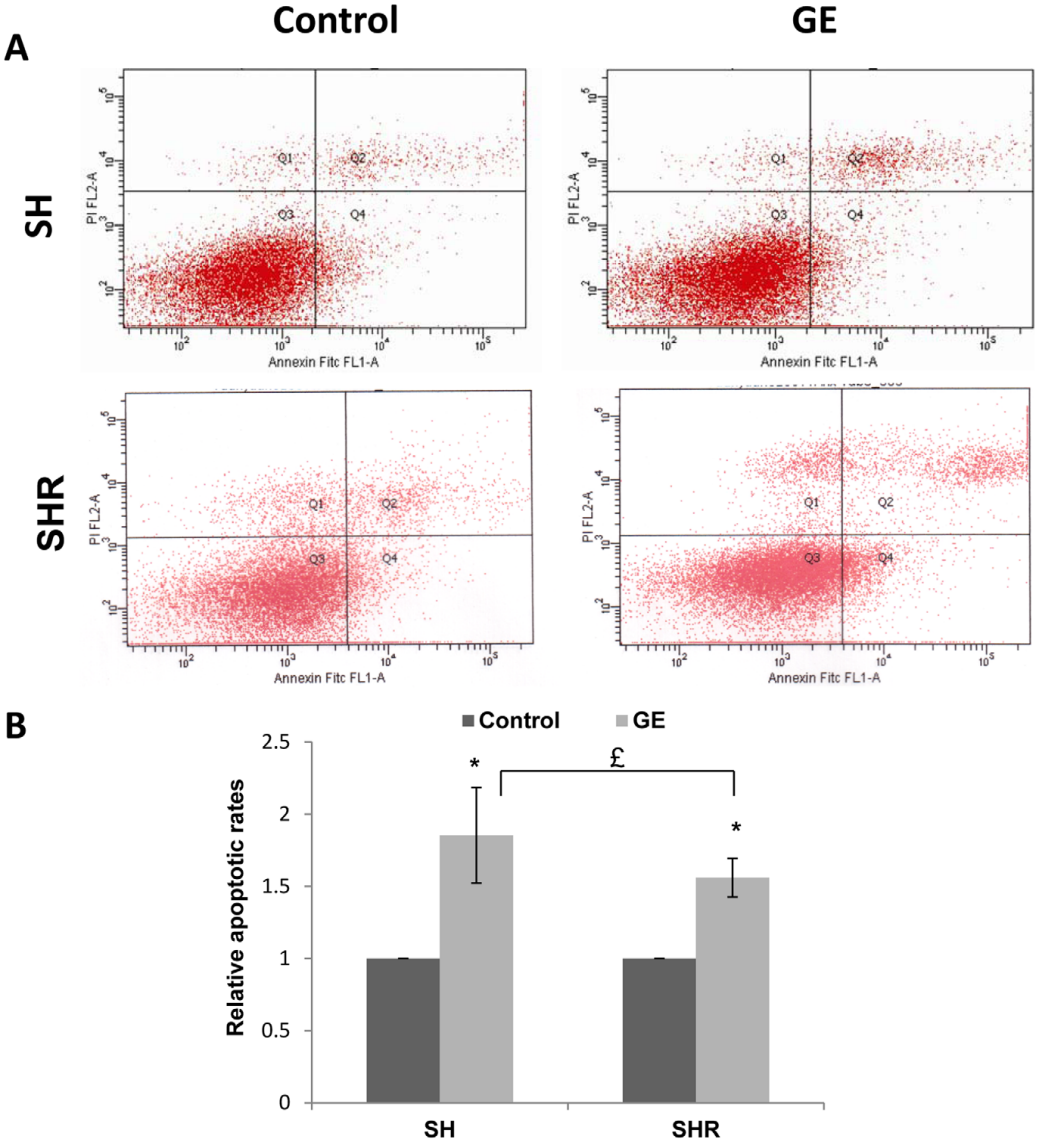
GE treatment results in apoptosis in transformed breast cells. A. Cell apoptosis of breast precancerous SH cells and transformed breast cancer SHR cells were detected by using an Annexin V and propidium iodide (PI) staining system following FACS-based flow cytometry assay. Cells were treated with 40 µM of GE for 3 days and harvested for apoptosis analysis. Apoptotic cells are shown in the upper right (as late apoptotic cells) or lower right (as early apoptotic cells) quadrants of the FACS histogram. B, Histogram of the apoptosis rate in SH and SHR cells in response to GE treatment. The relative apoptotic rate is the percentage of early plus late apoptotic cells normalized to levels of untreated samples. The graphs shown are representative of similar results obtained from three independent experiments. Columns, mean; Bars, SD; *, *P*<0.05, significantly different from control; £, *P*<0.05, significantly different from SHR cells.

To address how GE affects the proliferation of the cells, we performed cell apoptosis assays on precancerous SH cells and breast cancer SHR cells with the aforementioned treatment. We found that GE resulted in significant apoptosis in precancerous SH cells (fold change 1.85) and breast cancer SHR cells (fold change 1.56) compared to control cells as indicated in Fig. 2A and 2B. Consistent with the results of cell viability shown in Fig. 1, GE exhibited greater efficacy in inducing cellular apoptosis in pre-transformed SH cells than completely transformed SHR cells. These findings indicate that GE may more effectively exert its anti-cancer properties through reversal of breast tumorigenesis during initiation or at least very early in the process of tumorigenesis rather than later in the development of tumorigenic cells.

### GE treatment induced differential expression of tumor-related genes

**Figure 3:**
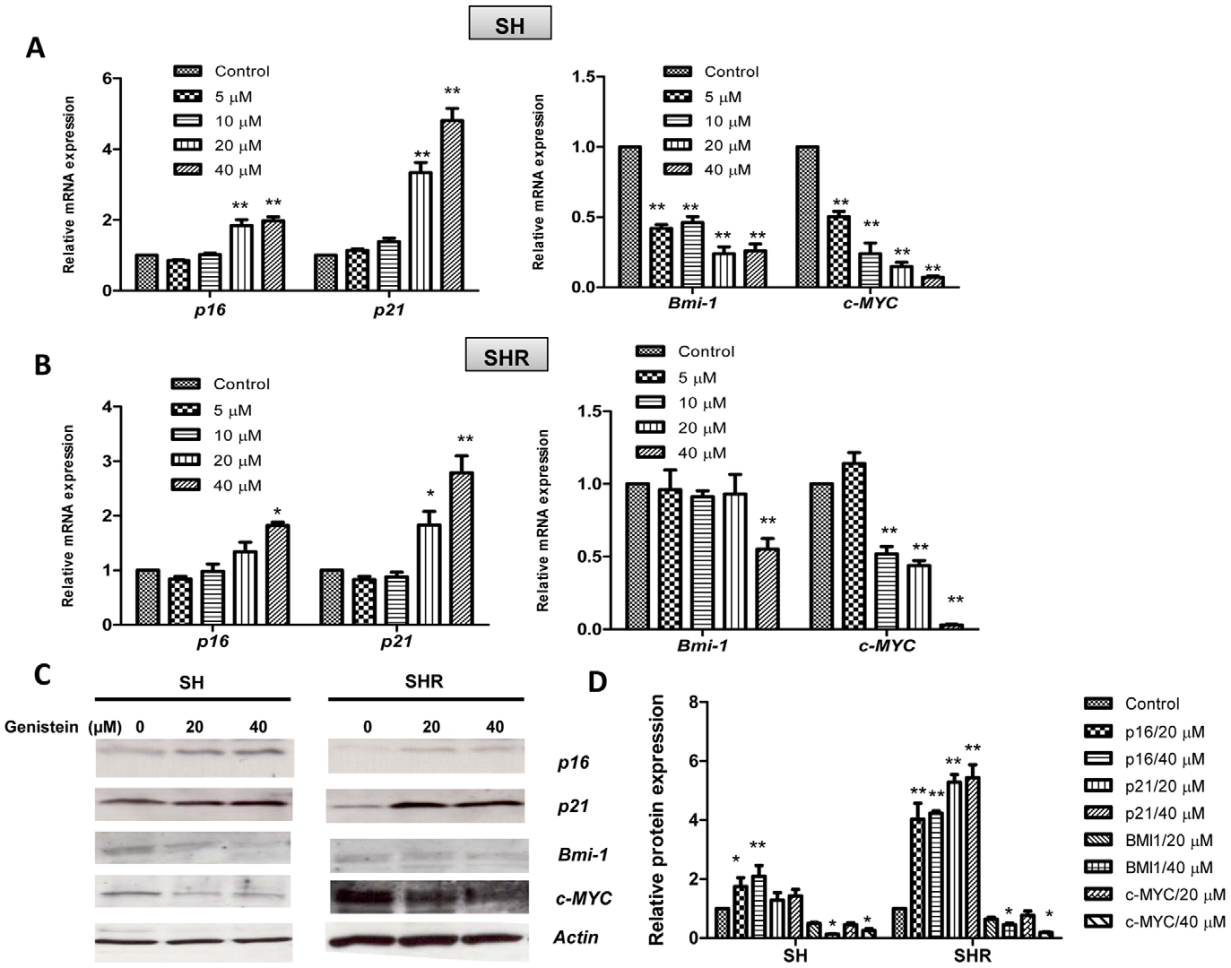
GE treatment induced differential expression of tumor-related genes. A and B mRNA expression changes of *p16, p21, BMI1* and *c-MYC* genes in breast precancerous SH cells (A) and completed transformed breast cancer SHR cells (B). Left panel, expression changes of tumor suppressor genes, *p16* and *p21*; right panel, expression changes of tumor promoting genes, *BMI1* and *c-MYC*. The cells were treated with various concentrations of GE for 3 days as described before. Control cells were grown in parallel with the treated cells but received vehicle DMSO. Quantitative real-time PCR was performed to measure relative transcription. C, The protein levels of p16, p21, BMI1 and c-MYC genes were determined by western-blot analysis. Actin antibody was used to ensure equal loading. Representative photograph from an experiment was repeated three times. D. Histogram of quantification of the protein levels. Data are in triplicate from three independent experiments and were normalized to *GAPDH* or Actin and calibrated to levels in untreated samples. Columns, mean; Bars, SD; *, *P*<0.01, * * *P*<0.001, significantly different from control.

We evaluated expression changes of several key epigenetic-regulated tumor-related genes including *p16^INK4a^* (*p16*), *p21^WAF1^* (*p21*), *BMI1* and *c-MYC*. Among these chosen genes, *p21* and *p16* are recognized as crucial tumor suppressor genes, whereas *BMI1* and *c-MYC* are important tumor promoting genes that can regulate expression of *p21* and *p16* as transcriptional factors [11], [35]–[37]. As shown in Fig. 3, GE treatment led to increased expression of tumor suppressor genes, *p16* and *p21*, whereas it decreased expression of tumor promoting genes, *BMI1* and *c-MYC*, in both precancerous SH (Fig. 3A) and breast cancer SHR cells (Fig. 3B) at the mRNA. These altered gene expressions exhibited a significant dose-dependent tendency. At the protein expression levels, we found that GE treatment led to significantly increased expression of p16 in both precancerous SH and breast cancer SHR cells (Figs. 3C and 3D). However, the protein level of p21 in SH cells was not significantly increased as we have observed its mRNA level in Figure 3A, suggesting a posttranslational regulation may be involved in GE-induced p21 regulation. Although we did not find significant changes in protein expressions of the BMI1 and c-MYC genes under 20 µM of GE treatment in SH and SHR cells, these two protein expressions showed significant repression at 40 µM of GE treatment as indicated in Figs. 3C and 3D. In addition, it is likely that GE at a relatively low concentration can result in more dramatic gene expression changes in pre-transformed SH cells than completely transformed SHR cells suggesting that GE may play a more important role to prevent breast cancer rather than treating it after it already occurs. These results are consistent with our previous results indicating an important preventive effect of GE during breast tumorigenesis, and GE-induced differential expression changes in these genes may be relevant to GE-associated breast cancer prevention.

### GE treatment led to alterations of histone modifications in the promoters of p21 and p16

**Figure 4:**
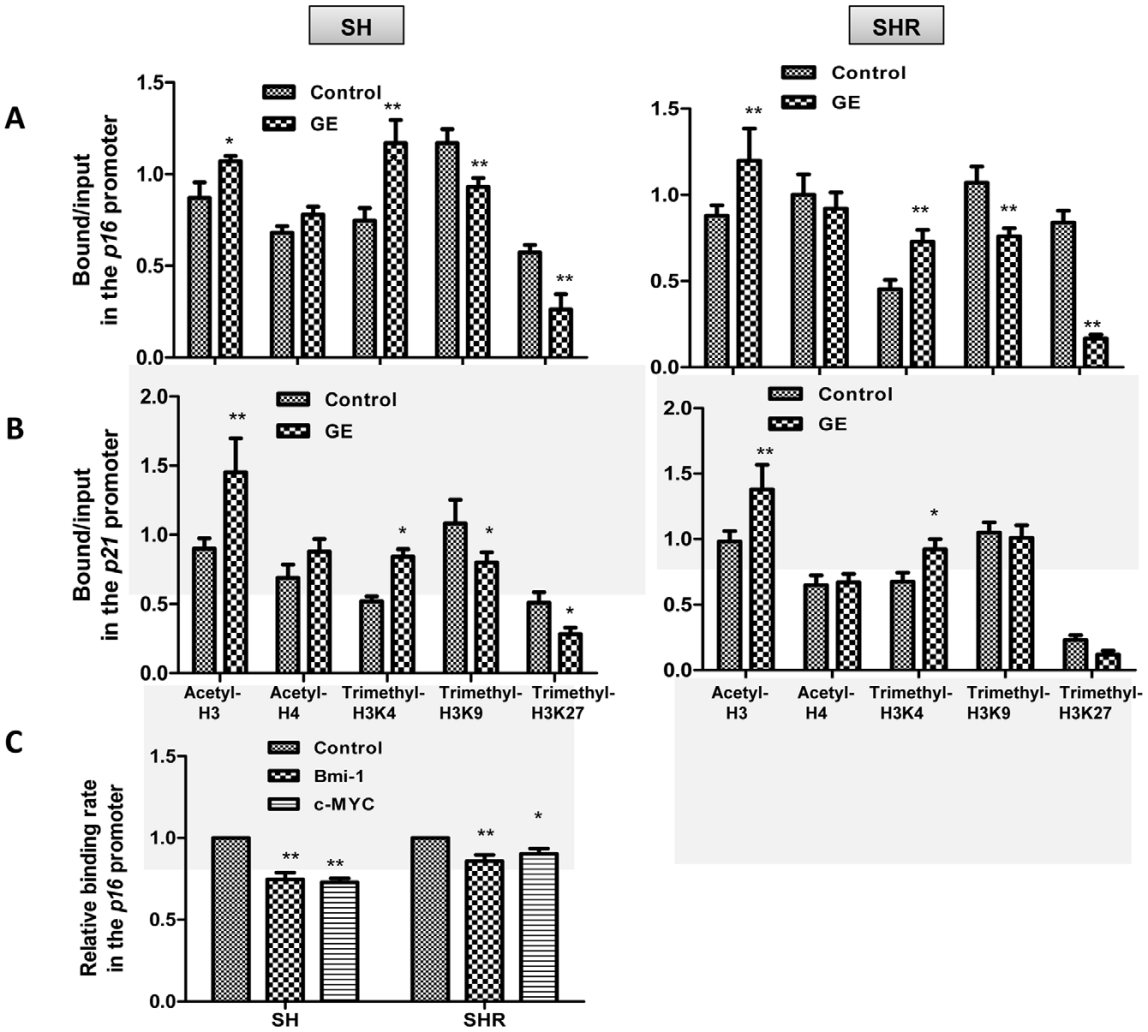
Histone modification alterations in response to GE treatment. A and B, Histone modification patterns in the promoters of *p16* (A) and *p21* (B) were analyzed by ChIP assay in breast precancerous SH cells (left panel) and completed transformed breast cancer SHR cells (right panel). Histone modification enrichment was calculated from the corresponding DNA fragments amplified by ChIP-PCR. The cells were treated with 40 µM of GE as described previously and analyzed by ChIP assays using chromatin markers including acetyl-H3, acetyl-H4, trimethyl-H3K4, trimethyl-H3K9, trimethyl-H3K27 and mouse IgG control in the promoter regions of *p16* and *p21*. C. Changes of binding abilities of *BMI1* and *c-MYC* in the *p16* promoter were determined by ChIP assay as described previously. Inputs came from the total DNA and served as the same ChIP-PCR conditions. DNA enrichment was calculated as the ratio of each bound sample divided by corresponding input. Data are in triplicate from three independent experiments. Columns, mean; Bars, SD; *, *P*<0.05, * * *P*<0.01, significantly different from control.

GE has been well documented as a bioactive dietary epigenetic modulator that regulates gene expression via affecting epigenetic pathways such as histone modifications, and *p16* and *p21* expression are frequently regulated by epigenetic factors during tumorigenesis [11], [12], [38]. To investigate the epigenetic effects of GE, we evaluated the patterns of histone modifications in the promoter regions of *p16* (Fig. 4A) and *p21* (Fig. 4B) in precancerous SH cells (left panel) and breast cancer SHR cells (right panel) by detecting several chromatin markers such as transcriptional active markers, acetyl-H3, acetyl-H4 and trimethyl-H3K4 as well as transcriptional repressor markers, trimethy-H3K9 and trimethyl-H3K27. Our results indicated that GE-induced chromatin changes correspondingly contribute to expression alterations of *p16* and *p21*. For example, GE treatment significant increased enrichment of chromatin activators, acetyl-H3 and trimethyl-H3K4, but decreased the binding of chromatin repressors, trimethy-H3K9 and trimethyl-H3K27, in the promoters of *p16* and *p21* of precancerous SH cells (Figs. 4A and 4B, left panel). Although the binding changes for chromatin markers of trimethyl-H3K9 and trimethyl-H3K27 in the promoter of *p21* were not promising in SHR cells, the relative changes in SH cells were significant. It suggests that both epigenetic and genetic mechanisms may play a role in regulation of GE-induced expression changes of *p16* and *p21* and both mechanisms may predominately regulate certain gene expressions which may be dependent on the stages of breast cancer development or different cell types.

These expression changes of key tumor-related genes may not be a direct consequence of epigenetic induction by GE treatment. However, it indicates a potential correlation between epigenetic regulation and GE-induced key gene expression changes that is associated with preventive and therapeutic effects of GE on breast cancer.

### GE inhibited recruitment of repressor complex on the p16 promoter

To our knowledge, *c-MYC* and its co-factor, *BMI1*, as key transcription factors, are responsible for regulating the expression of key tumor-related genes such as *p16*. A possible mechanism for the collaboration of *c-MYC* and *BMI1* was suggested by studies demonstrating that *BMI1* inhibits *c-MYC*-induced apoptosis through *p16* repression [39]. We therefore conducted experiments to investigate the potential collaborative effects of *c-MYC* and *BMI1* on transcriptional regulation of *p16* and *p21* expression in response to GE treatment. As shown in Fig. 4C, GE treatment significantly impaired binding abilities of the *c-MYC* and *BMI1* to the *p16* promoter, indicating GE may reverse *p16* expression by inhibiting recruitment of transcriptional repressor complex such as BMI1-c-MYC complex. However, we failed to detect binding of *c-MYC* and *BMI1* in the *p21* promoter (data not shown) suggesting that *p21* may not be a direct transcriptional target for the BMI1-c-MYC repressor complex.

### Epigenetic enzymatic activity changes in response to GE

**Figure 5:**
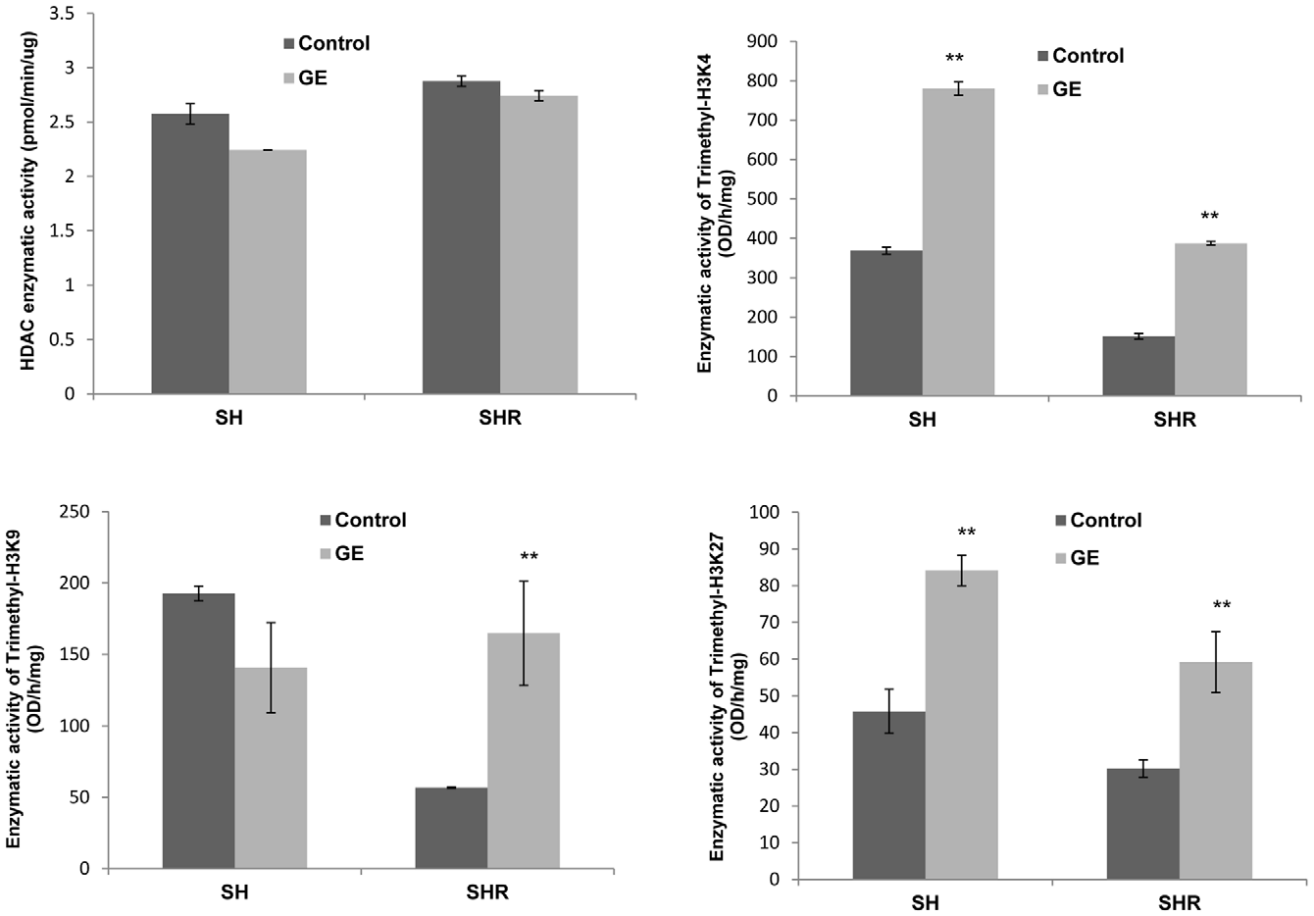
Epigenetic enzymatic activity changes in response to GE treatment. A, HDACs enzymatic activity; B, HMT (trimethyl-H3K4) activity; C. HMT (trimethyl-H3K9) activity; D. HMT (trimethyl-H3K27) activity. Nuclear proteins of SH and SHR cells were extracted after the treatment as described above. The enzymatic activity assays were performed according to the manufacturer's protocols. The values of enzymatic activities came from the means of three independent experiments. Columns, mean; Bars, SD. **, *P*<0.01, significantly different from control.

To further interpret the mechanisms of epigenetic modulations by GE treatment during breast tumorigenesis, we assessed several important epigenetic enzymatic activities including enzymes involved histone acetylation such as histone deacetylases (HDACs) as well as histone methylation such as histone methyltransferases (HMTs) (trimethyl-H3K4, trimethyl-H3K9 and trimethyl-H3K27). As shown in Fig. 5A, GE treatment slightly reduced HDACs activity in both SH and SHR cells, which is consistent with our previous studies on other types of breast cancer cells [40]. To the contrary, GE induced overwhelming enzymatic activation in most of the tested HMTs (Figs. 5B, 5C and 5D), which control gene expression through chromatin-dependent transcriptional repression or activation [41]. These results indicate that GE may affect epigenetic pathways most likely via influencing histone methylation, which could be an important contributor affecting expression of key tumor-related gene such as *p16* and *p21* through direct or indirect mechanisms.

### Dietary GE inhibited the growth of breast cancer xenografts

To determine the *in vivo* breast cancer inhibitory properties of GE, we conducted animal experiment to examine whether dietary administration of GE can inhibit the growth of breast cancer xenografts. Although precancerous SH cells obtained the ability to grow immortalized, these cells were proven failed to generate tumors in nude mice [26]. We therefore used transformed breast cancer cells, SHR, to grow xenografts in athymic nude mice that had been fed a diet supplemented with GE for two weeks before injection of the tumor cells and continued throughout the study. The mice were given the GE diet at a concentration of 250 mg/kg, which is considered physiologically available compared to human daily consumption of soybean products based on the previous studies [29], [40].

**Figure 6:**
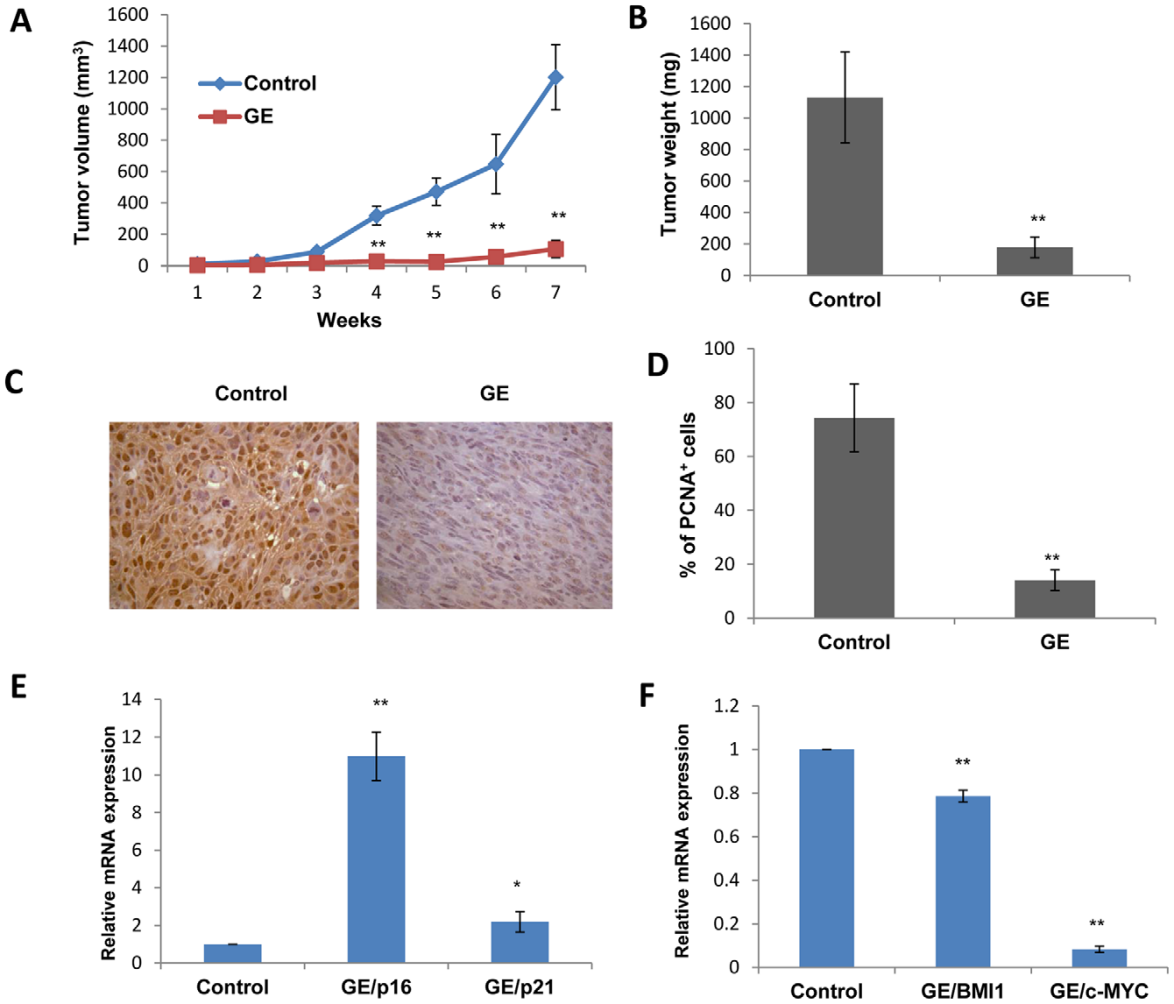
Breast tumor growth in a mouse xenograft model by dietary GE treatment. Female athymic nude mice were injected with transformed breast cancer SHR cells. GE or control diets were provided from two weeks prior to injection and continued throughout the study. A, Tumor volume during the experiment. B. Tumor weights when xenograft tumors were harvested at the termination of the experiment. Tumor volumes were calculated by using the formula: volume (mm^3^)  =  (length × width^2^) ×0.523, and represented as mean ± SD (mm^3^) for each group. Tumor weight is the wet weight of the tumor per mouse in each group and is reported as mean ± SD (g); C. PCNA expression in SHR tumor xenogratfs. Immunohistochemical analysis was performed in tumor samples to detect PCNA-positive cells for proliferation index. D. Graphic representation of expression. Immunohistochemical data in terms of percentage of positive cells are presented as mean ± SD from each group. PCNA-positive cells were counted in 5 different areas of the sections, and data are summarized in terms of percent positive cells from all tumor samples. Representative photograph from one field of each experimental group. E. Expression changes of tumor suppressor genes, *p16* and *p21* in mice SHR xenograft tumors; F. Expression changes of tumor promoting genes, *BMI1* and *c-MYC* in mice SHR xenograft tumors. Symbols and columns, mean; Bars, SD; * *p*<0.01, **, *p*<0.001 significantly different from control group.

Periodic measurement of the tumor volume indicated that dietary GE completely suppressed tumor growth throughout the whole experimental timeframe compared with the control group (Fig. 6A) suggesting GE may interfere with breast tumor growth from the very early stages of breast tumor initiation. The wet weight of the SHR xenograft tumors per mouse was significantly lower in the mice administered the GE diet than in the mice that did not receive GE in diet (*p*<0.01, Fig. 6B). The above data have been further interpreted in Table 1 indicating striking effects of GE on prevention (prevention rate 60%) as well as repression of breast tumor growth (inhibition rate 86.25%). We also analyzed the potential *in vivo* anti-proliferative property of GE administration by detecting PCNA-positive cells in mice SHR xenograft tumors using immunohistochemical assays. As shown in Figs. 6C and 6D, PCNA-positive cells that represent proliferating cells in mice xenograft tumors were significantly depleted, and the percentage of PCNA-positive cells were significantly reduced by 5.23 fold in breast tumors of the GE group as compared to the control group. This result indicates that dietary GE can inhibit breast tumor growth in mouse *in vivo* analysis by affecting the proliferation rate of tumor growth. Since the aforementioned studies indicated that GE treatment induced differential expression of key tumor-related genes *in vitro*, we sought to further investigate whether dietary GE can impact a similar mechanism *in vivo*. We evaluated mRNA expression of *p16, p21, BMI1* and *c-MYC* in mice SHR tumor xenografts. As shown in Fig. 6C, mRNA levels of two tumor suppressor genes, *p16* and *p21*, were significantly increased (*p*<0.01, Fig. 6E), whereas the expression of two tumor promoting genes, *BMI1* and *c-MYC*, were dramatically decreased (*p*<0.01, Fig. 6F) in response to GE treatment. These results are consistent with our aforementioned *in vitro* studies indicating the important role of these tumor-related genes that may contribute to GE-induced breast tumor prevention and early suppression during breast tumorigenesis.

**Table 1:** GE inhibited tumor growth in mouse SHR xenografts.

Animal Group	Diet and treatment	^a^ Prevention rate (%)	TV^b^ (mm^3^ mean ± SD)	RTV^c^ (mean )	IR^d^ (%)
**Control**	Modified AIN-93G diet 7% of corn oil instead of soy oil.	0	1201.195±807.04	60.06	-
**GE**	GE diet contains 250 mg genistein/kg of modified AIN-93G diet.	60	165.143±357.516	8.26	86.25

a. Prevention rate  =  tumor-free mice number/total mice number; b. Tumor volume (TV)  =  (length × width^2^) ×0.532; c. Relative Tumor volume (RTV)  =  (TV on sacrificing day)/(TV on day 1 of injection); d. Inhibition rate on tumor growth (IR)  =  {1 – (mean RTV of the treatment group)/(mean RTV of the control group)} 100.

## Discussion

Breast tumorigenesis is a complicated pathological process that involves a series of aberrant expressions in various tumor-suppressor genes and oncogenes due to, at least in part, genetic and epigenetic abnormalities during early tumor initiation. We designed our studies to better understand the potential molecular mechanisms during breast tumorigenesis, and more importantly, to explore the preventive properties of a bioactive dietary compound, soybean genistein (GE), in reversing breast malignancy at its early stages.

We started our work on dietary GE since GE is known as an effective anti-breast cancer compound and can impact gene expression through epigenetic regulations. An established cancer chemoprevention model that causes normal breast cells to undergo cancer initiation was used in this study. This transformation model system was originally developed by Weinberg and his colleagues in 1999, which has been accomplished oncogenic transformation through the viral-mediated serial gene transfer of three defined elements, *SV40, hTERT* and *hRAS-V12*, to normal human epithelial cells [25], [26] Although these individual genetic mutations are not universally found in all types of breast cancer, the model has been widely used for breast cancer research since it can closely approximate the initiation and early progression of breast cancer [27], [42]. We therefore feel that this model enables us to assess the impact of the GE in real-time not only in preventing the transition of oncogenesis, but in preventing the early epigenetic aberrations that are frequently associated with cancer. To our knowledge, this is the first chempreventive application of this model system.

Strikingly, our results showed that GE induced more significant effects on cellular viability inhibition and apoptotic response in precancerous SH than in completed transformed breast cancer SHR cells, which suggests for the first time that GE may predominately employ its anti-cancer effect at an early stage of breast tumorigenesis. Cancer development is driven by the sequential abnormalities of gene expression profiles, such as the constitutive activation of oncogenes and the loss of function of tumor-suppressor genes. Our further results showed that GE treatment induced a differential expressional pattern of several tumor-related genes, including increased expression of two crucial tumor suppressor genes, *p21* and *p16*, and decreased expression of two tumor promoting genes, *BMI1* and *c-MYC*. As key tumor suppressor genes, *p21* and *p16*, known to induce cell cycle arrest leading to growth inhibition in various tumor cell lines, are frequently silenced due in part to aberrant epigenetic regulations [12], [38]. *BMI1* and *c-MYC* are identified as proto-oncogenes that function as transcriptional repressors that silence specific sets of genes and promote tumorigenesis [36], [37]. Consistently, our further *in vivo* studies revealed that orally-administered GE can significantly inhibit the tumor growth of breast xenografts in orthotopic mouse models through, at least in part, regulation of these gene expressions. Therefore, these genes play an important role during the early stages of breast tumor development and the differential expression profiles of these tumor-related genes may contribute to GE-induced breast cancer prevention and interference with early cancer development.

The compaction of the chromatin state is closely related to patterns of gene expression, which is regulated in part by a complex array of post-translational modifications of histones [8]. Altered histone modification codes lead to perturbations of chromatin structure that can cause inappropriate gene expression and genomic instability, resulting in cellular transformation and malignant initiation [6], [7]. GE has been known as a bioactive dietary epigenetic modulator regulating gene expression via affecting epigenetic pathways such as histone modifications. Our results indicate that GE altered the status of histone acetylation and methylation in the promoters of *p16* and *p21*, which may contribute to increased expression of these two genes by GE. We also noticed that this effect was likely more prominent in precancerous SH cells rather in breast cancer SHR cells, further suggesting that GE may exert its anti-cancer properties in the early stages of breast cancer development and epigenetic mechanisms may be important in the preventive efficacy of GE. Although the epigenetic changes are not promising in breast cancer SHR cells, it is possible that genetic mechanisms may play a more important role in regulation of GE-induced key tumor-related gene expression in completely transformed breast cancer cells. Our further evaluations of epigenetic enzymatic activities indicate that GE-induced global activation in HMTs could be a crucial factor contributing to its epigenetic modulator effects on regulation of key tumor-related gene expressions.

Recent studies demonstrated an important gene repressive mechanism involving recruitment of several transcriptional repressors such as the polycomb group protein, *BMI1*, to certain locally methylated histones such as trimethylation (me3) of lysine 27 of target genes including *p16* [36], [43]. Previous studies also indicated that *BMI1* can cooperate with *c-MYC* to repress *p16* expression in the regulation of cellular proliferation during tumorigenesis [39], [44]. Our finding revealed that GE interfered with the binding of the transcriptional repressor complex, BMI1-c-MYC to the *p16* promoter. Combined with the aforementioned results for GE regulation of histone modification patterns, our results suggest a novel mechanism for GE-induced breast tumorigenesis inhibition. In this model, GE may inhibit recruitment of BMI1-c-MYC repressor complex to the regulatory region of the *p16* promoter by down-regulation of *c-MYC* and *BMI1* expression and impairing binding abilities of the transcriptional repressive complex by influencing histone markers throughout the recognized chromodomain.

The present study provides the first insights into the possible epigenetic mechanisms underlying the function of GE in inhibition of breast tumorigenesis. The results imply a dynamic role for GE in control of several tumor-related genes through an epigenetic mechanistic pathway. It is intriguing that the prevention properties of GE function have been linked to key gene expression regulation through epigenetic control. In this regard, it may provide a novel preventive and therapeutic approach targeting selective gene therapy through consumption of a natural dietary ingredient, GE, combined with conventional epiegentic therapies. Future research will undoubtedly unravel the specific contributions of GE in breast cancer prevention and therapy by testing its efficacy in human clinical trials that will lead the applicability of these novel approaches.

## Supporting Information

Information S1 **Dose-dependent inhibition rates and IC50 by GE treatment in transformed breast cells.** A, B, C, Dose-dependent inhibition rates in breast precancerous SH cells (A), transformed breast cancer SHR cells (B) and breast cancer MDA-MB-231 cells (C) were determined by MTT assay. Cells were treated with various concentrations of GE in a 96-well plate for 72 h. Dose-dependent inhibition rates and IC50 were analyzed by non-linear regression analysis. D. Table summary for IC50 values.
(TIF)
