# Supplementary figures and images for: Epigenetic Regulation of Multiple Tumor-Related Genes Leads to Suppression of Breast Tumorigenesis by Dietary Genistein
Source: PLoS One. 2013 Jan 14;8(1):e54369. doi: 10.1371/journal.pone.0054369 (PMC3544723; doi:10.1371/journal.pone.0054369)

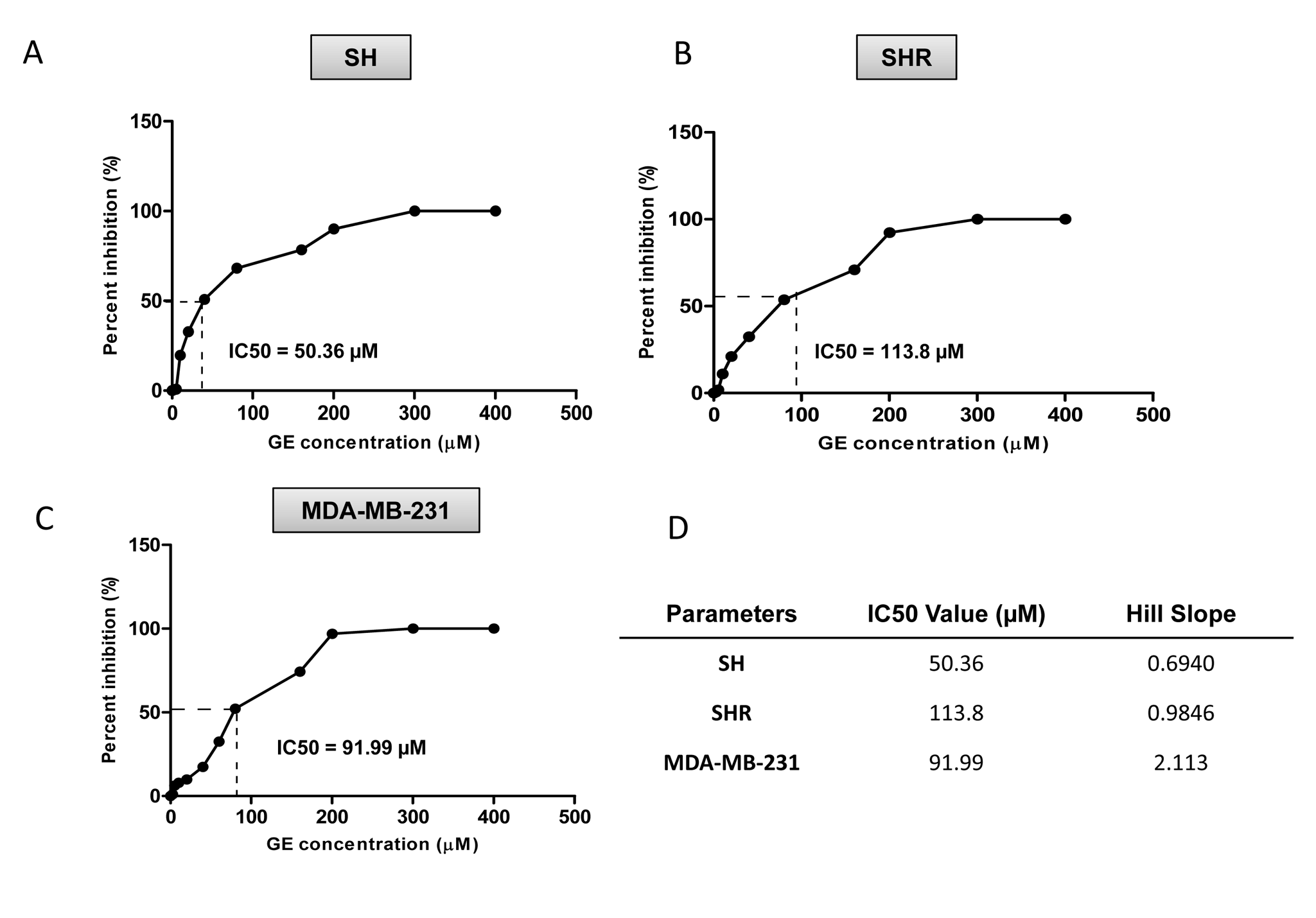

Supplement: Information S1 — Dose-dependent inhibition rates and IC50 by GE treatment in transformed breast cells. A, B, C, Dose-dependent inhibition rates in breast precancerous SH cells (A), transformed breast cancer SHR cells (B) and breast cancer MDA-MB-231 cells (C) were determined by MTT assay. Cells were treated with various concentrations of GE in a 96-well plate for 72 h. Dose-dependent inhibition rates and IC50 were analyzed by non-linear regression analysis. D. Table summary for IC50 values. (TIF) [file pone.0054369.s001.tif]
